# Diagnosis of primary squamous cell carcinoma of the pancreas using endoscopic ultrasound-guided core needle biopsy

**DOI:** 10.1093/gastro/gov018

**Published:** 2015-05-16

**Authors:** Amir Kashani, Melissa Kahn, Laith H. Jamil

**Affiliations:** 1 Division of Gastroenterology, Cedars-Sinai Medical Center, Los Angeles, USA; 2 Department of Pathology and Laboratory Medicine, Cedars-Sinai Medical Center, Los Angeles, USA

**Keywords:** squamous cell carcinoma, pancreatic neoplasms, endoscopic ultrasonography, core needle biopsy

## Abstract

Primary squamous cell carcinoma (SCC) of the pancreas is a particularly rare entity. Diagnosis of this tumor is tentatively made after ruling out metastatic SCC from another primary site and adenosquamous carcinoma (ASC) of the pancreas. Here we discuss the case of a 76-year-old woman who was found to have a solitary pancreatic lesion and multiple hepatic lesions. Results of computed tomography-guided biopsy of the liver lesions were consistent with a metastatic carcinoma displaying squamous differentiation; therefore, an endoscopic ultrasound (EUS)-guided core-needle biopsy (CNB) of the pancreatic mass was performed. Meticulous histopathological examination of the pancreatic specimen at multiple levels revealed moderately well-differentiated SCC with no glandular component. An extensive metastatic work-up did not reveal an extra-pancreatic origin for this SCC; hence, a diagnosis of primary SCC of the pancreas was established. To our knowledge, this is the first report of the diagnosis of a primary SCC of the pancreas using EUS-guided CNB. We believe that CNB has a diagnostic yield equivalent to that of fine-needle aspiration for recognizing pancreatic adenocarcinoma; however, when cytological examinations reveal atypical squamous epithelial cells suggestive of malignancy, CNB may provide a better tissue specimen, from which to determine the presence of a glandular component. Such an assessment will differentiate pancreatic SCC from ASC.

## Introduction

Primary squamous cell carcinoma (SCC) of the pancreas is a particularly rare tumor that has been suggested to originate from the pancreatic ductal cells [1]. Since native pancreatic tissue lacks squamous epithelium, finding these cells in a pancreatic tumor raises the possibility of metastatic disease from another primary site [2]. Diagnosis of a primary pancreatic SCC is made only after metastatic disease and adenosquamous carcinoma (ASC)—another rare primary tumor of the pancreas—has been excluded [2]. Herein we report our experience in diagnosing a primary SCC of the pancreas; also, we will elaborate on the utility of endoscopic ultrasound (EUS)-guided core-needle biopsy (CNB) in our diagnostic approach.

## Case Presentation

**Figure 1.**
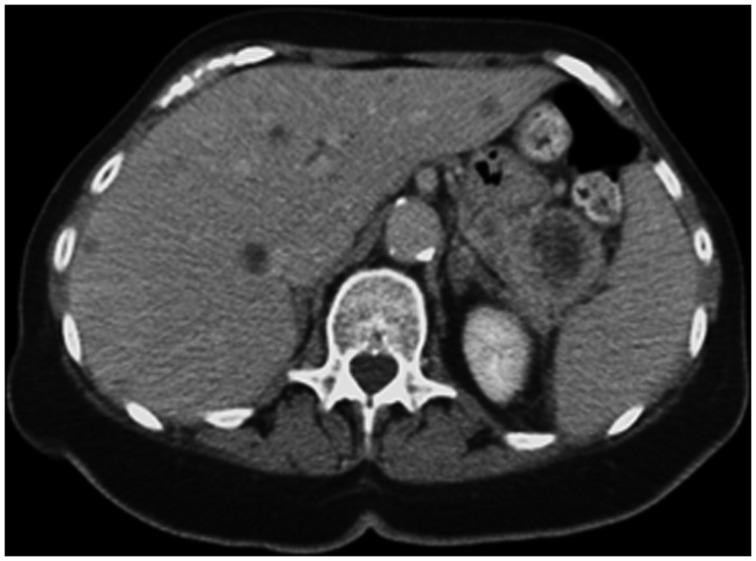
Abdominal computed tomography reveals a 5 × 3.7 cm pancreatic-tail mass with central necrosis and multiple lesions throughout the liver, with central low attenuation.

**Figure 2.**
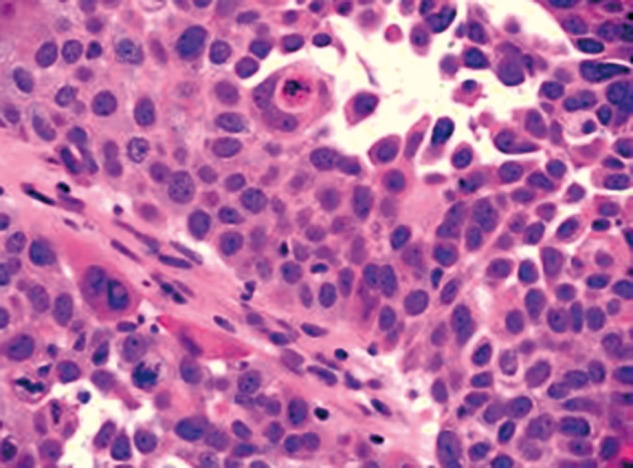
Histological characteristics of the liver specimen are consistent with metastatic carcinoma displaying squamous differentiation (hematoxylin and eosin stain, original magnification ×400).

A 76-year-old woman with a medical history of Alzheimer’s disease was admitted to our institution with generalized weakness and leukocytosis (17 700 cells/mL; reference value: 4 000–11 000 cells/mL). To investigate the origin of the leukocytosis, an abdominal computed tomography (CT) scan was performed, which revealed a large pancreatic-tail mass in addition to multiple hepatic lesions (Figure 1). Findings on endoscopic evaluation of the upper and lower gastrointestinal tract were unremarkable. A CT-guided biopsy of one of the liver masses was then performed; the histopathology was consistent with a metastatic carcinoma displaying squamous differentiation (Figure 2). Immunohistochemical studies were positive for cytokeratin 5/6, cytokeratin AE1/AE3, and p63 and negative for CDX2. Significant elevation of serum tumor markers, including carbohydrate antigen 19-9 (846 U/mL; reference value: <37 U/mL), cancer antigen 125 (1686 U/mL; reference value: <35 U/mL), and carcinoembryonic antigen (170 ng/mL*; *reference value: <3 ng/mL), was noted.

**Figure 3.**
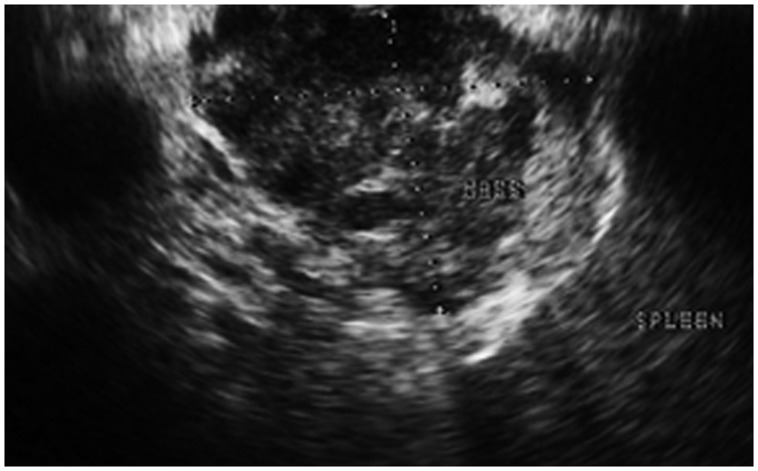
Endoscopic ultrasonography shows the pancreatic-tail mass, with mixed echogenicity and areas with cystic components suggestive of necrosis.

**Figure 4.**
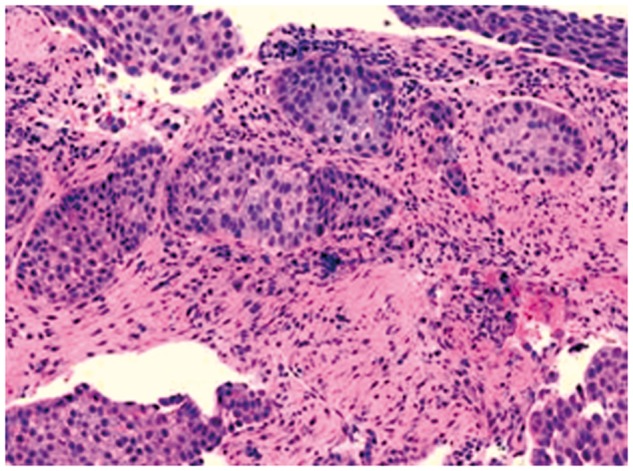
Histological characteristics of the pancreatic mass are consistent with moderately well-differentiated squamous cell carcinoma, with no glandular pattern (hematoxylin and eosin stain, original magnification ×100).

With the origin of this metastatic SCC unclear, a biopsy of the pancreatic mass was undertaken with endoscopic ultrasound (EUS) guidance (Figure 3). Introducing a 22-gauge needle, fine-needle aspiration (FNA) was performed, with eight passes. Onsite evaluation by a cytopathologist revealed a few highly atypical cells. Thereafter, applying a Quick-Core® needle (Cook Medical Inc., Bloomington, IN, USA), EUS-guided CNB of the mass was performed, with three passes. Cytology showed scattered large sheets and clusters of malignant epithelial cells with enlarged pleomorphic nuclei, vesicular chromatin, prominent nucleoli, and a moderate amount of cytoplasm with no frank cytoplasmic keratinization. Histological examination of the core specimen was consistent with moderately well-differentiated SCC (Figure 4); the specimen did not contain any glandular component, nor did mucicarmine staining reveal mucin.

**Figure 5.**
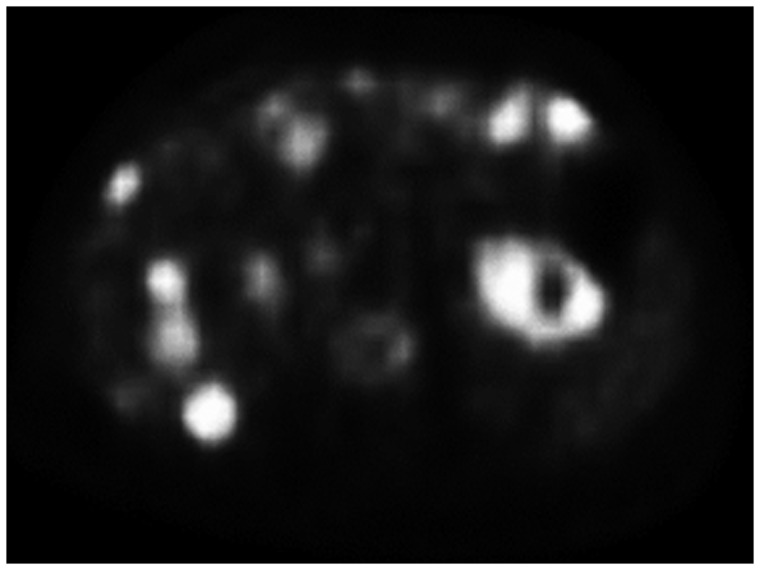
Positron emission tomography reveals a large, intensely metabolically active pancreatic-tail mass with a central photopenic area, consistent with a neoplastic lesion with central necrosis. Also visible are extensive, intensely metabolically active, low-density lesions scattered throughout the liver, consistent with liver metastatic involvement.

Although the imaging and endoscopic work-up did not reveal any extra-pancreatic origin for SCC, a whole-body 18[F]-fluorodeoxyglucose positron emission tomography/CT (FDG-PET/CT) scan was conducted; this showed increased metabolic activity only in a focal area of the pancreas and multiple areas throughout the liver (Figure 5). These findings confirmed the diagnosis of primary SCC of the pancreas with liver metastasis.

## Discussion

Because it is a rare entity, a diagnosis of primary pancreatic SCC is made only after excluding other neoplastic lesions of the pancreas that contain squamous epithelial components [2]. The main differential diagnoses of primary SCC of the pancreas are ASC and metastatic SCC from another primary site [3]. To exclude ASC of the pancreas, meticulous histopathological examination of the pancreatic specimens at several cuts is crucial, the purpose being to look for glandular components in the specimen [4]. The presence of glandular tissue alongside squamous epithelium confirms the diagnosis of ASC [2]; these characteristics are very unlikely to be found in metastatic disease of the pancreas.

Tissue adequacy is essential for a thorough pathological examination. Minimally invasive methods, such as FNA, are replacing traditional surgical approaches for obtaining pancreatic tissue [5]. These methods do not always provide adequate tissue to exclude glandular components with high certainty, limiting the ability of the pathologist to differentiate ASC from SCC [2]. In the present case, an EUS-guided CNB was used in diagnosis of a primary SCC of the pancreas. In previous studies, the diagnostic yield of CNB was found to be no higher than that of FNA for pancreatic lesions [6]. However, the common pathological condition evaluated in those studies was adenocarcinoma of the pancreas. The presence of squamous epithelium in a specimen retrieved through FNA will necessitate whole-tissue examination to exclude the co-existence of a glandular component. It is possible that, on the initial evaluation using FNA, the only finding is a squamous component; while later examination of a whole-tissue specimen may also reveal a glandular component [7]; such a finding could change the diagnosis from SCC to ASC. In the present case, only after a thorough examination of the whole-tissue specimen retrieved by EUS-guided CNB was the pathologist able to diagnose SCC.

Aspiration of the specimen through the skin—or even via the endoscopic route—may cause contamination of the squamous epithelium of other organs, such as skin or the esophagus [2, 8]. It has been shown that the possibility of contamination is higher when the specimen is acquired by FNA than when obtained by CNB [6]. Previous inflammatory conditions, such as pancreatitis or pancreatic duct stenting, may lead to squamous metaplasia and the presence of these cells in an FNA specimen [2]. Although contaminants in the specimen are generally recognizable by a lower number of atypical cells and degree of nuclear atypia, squamous metaplastic epithelium shows greater similarity to malignancy [2].

Once a diagnosis of SCC is established, the challenge is to differentiate primary- from metastatic SCC of the pancreas. To establish a tumor as primary, an extensive metastatic work-up is warranted [1]. Notably, histopathological studies are not capable of differentiating metastatic from primary pancreatic SCC [4]. Performing an FDG-PET/CT scan has been introduced as a method of locating the origin of an SCC [9]. The pancreas is generally diffusely involved in the case of metastatic disease and isolated metastatic lesions of the pancreas have rarely been reported [2]. In our case, an FDG-PET/CT scan revealed a solitary pancreatic lesion in addition to multiple hepatic lesions; an endoscopic work-up, along with other imaging studies, did not suggest any other sites as primary; therefore, we could confidently diagnose our patient as having a primary pancreatic SCC with metastasis to the liver.

In conclusion, we described our systematic diagnostic approach in evaluating a case of rare primary pancreatic SCC. When cytological examination of a pancreatic tumor reveals atypical squamous epithelium suggestive of malignancy and surgical tissue acquisition is not an option, we suggest performing an EUS-guided CNB of the lesion. This may provide a better tissue specimen, enabling a more thorough examination to determine the presence of a glandular component to differentiate pancreatic SCC from ASC. An extensive metastatic work-up, including a whole-body FDG-PET/CT scan, is deemed essential to evaluate the possibility of metastatic *vs*. primary pancreatic SCC.

*Conflict of interest statement:* none declared.
